# Impact of COVID‐19 pandemic lockdown on narcolepsy type 1 management

**DOI:** 10.1002/brb3.1955

**Published:** 2020-11-28

**Authors:** Emanuela Postiglione, Fabio Pizza, Francesca Ingravallo, Luca Vignatelli, Marco Filardi, Anastasia Mangiaruga, Elena Antelmi, Monica Moresco, Claudia Oriolo, Uberto Pagotto, Giuseppe Plazzi

**Affiliations:** ^1^ Department of Biomedical and Neuromotor Sciences (DIBINEM) University of Bologna Bologna Italy; ^2^ IRCCS Istituto delle Scienze Neurologiche di Bologna Bologna Italy; ^3^ Department of Medical and Surgical Sciences (DIMEC) University of Bologna Bologna Italy; ^4^ Neurology Unit Movement Disorders Division Department of Neurosciences, Biomedicine and Movement Sciences University of Verona Verona Italy; ^5^ Endocrinology and Diabetes Prevention and Care Unit Department of Medical and Surgical Sciences (DIMEC) S. Orsola Policlinic University of Bologna Bologna Italy; ^6^ Department of Biomedical, Metabolic and Neural Sciences University of Modena and Reggio‐Emilia Modena Italy

**Keywords:** behavioral therapy, COVID‐19, lockdown, narcolepsy type 1, smart working

## Abstract

**Study Objectives:**

Narcolepsy type 1 (NT1) is a chronic rare hypersomnia of central origin requiring a combination of behavioral and pharmacological treatments. During the coronavirus disease 2019 (COVID‐19) pandemic, in Italy the population was forced into a lockdown. With this study, we aimed to describe the lockdown impact on NT1 symptom management, according to different patients' working schedule.

**Methods:**

In the period between 10 April and 15 May 2020, we performed routine follow‐up visits by telephone (as recommended during the COVID‐19 emergency) to 50 patients >18 years old (40% males) under stable long‐term treatment. We divided patients into three groups: unchanged working schedule, forced working/studying at home, and those who lost their job (“lost occupation”). Current sleep–wake habit and symptom severity were compared with prelockdown assessment (six months before) in the three patient groups.

**Results:**

At assessment, 20, 22, and eight patients belonged to the unchanged, working/studying at home, and lost occupation groups, respectively. While in the lost occupation group, there were no significant differences compared with prepandemic assessment, the patients with unchanged schedules reported more nocturnal awakenings, and NT1 patients working/studying at home showed an extension of nocturnal sleep time, more frequent daytime napping, improvement of daytime sleepiness, and a significant increase in their body mass index. Sleep‐related paralysis/hallucinations, automatic behaviors, cataplexy, and disturbed nocturnal sleep did not differ.

**Conclusions:**

Narcolepsy type 1 patients working/studying at home intensified behavioral interventions (increased nocturnal sleep time and daytime napping) and ameliorated daytime sleepiness despite presenting with a slight, but significant, increase of weight.

## Graphical abstract

The graphical abstract displays that NT1 patients working/studying at home slept more with improved subjective daytime sleepiness, while both NT1 patients working/studying at home and loosing occupation because of the pandemic significantly increased BMI.

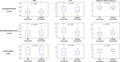

## 1 INTRODUCTION

Narcolepsy type 1 (NT1) is a rare, chronic, disabling disease. Daytime sleepiness, automatic behaviors, and nocturnal sleep alterations (i.e., disrupted nocturnal sleep, hypnagogic hallucinations, and sleep paralysis) are common symptoms. Metabolic and psychiatric comorbidities contribute to seriously affecting patients' quality of life (Raggi et al., 2019). Therefore, disease management requires highly specialized and multidisciplinary medical care.

Currently available therapeutic approaches for NT1 are symptomatic and include a combination of behavioral and pharmacological interventions carefully targeted on patient needs at different ages, taking into account daily scheduled routines (schooling, work, family management, etc.). A close follow‐up is therefore needed to keep up with the changing needs and to promptly modulate therapeutic interventions in order to minimize disease burden (Maski et al., 2017). In Italy, the Narcolepsy Centre of Bologna, located in the Emilia‐Romagna region, represents the national center of reference (more than 70% of the assisted patients come from outside the Emilia region) and offers a unique multidisciplinary approach, that is, encompassing sleep, metabolic, psychological, and psychosocial aspects.

Between February and March 2020, Italy was the country with the most cases and deaths related to the coronavirus disease 2019 (COVID‐19) pandemic, and the first one forced to adopt lockdown measures in Europe, initially restricted only to the northern regions (Li et al., 2020). Starting from 11 March 2020, after the closure of schools, the Italian government established a national lockdown with a rapidly progressive restriction of personal mobility up to in‐home confinement, that is, limiting the movement of the population except for necessity, work, and severe health circumstances (https://www.gazzettaufficiale.it/eli/id/2020/03/11/20A01605/sg).

To guarantee care continuity, the Local Health Trust of Bologna encouraged neurologists from the Narcolepsy Centre of Bologna to perform telephone follow‐up consultations to limit inhospital visits and only allow them for patients requiring urgent or essential visits. This allowed us to record the impact of the lockdown on working schedules, sleep–wake patterns, and symptom severity of NT1 patients under long‐term stable pharmacological treatment.

## 2 MATERIALS AND METHODS

### 2.1 Methods

We included all consecutive patients who agreed to undergo a preplanned follow‐up telephone neurologic consultation from 16 April to 12 May 2020, in the middle of the Italian lockdown related to the COVID‐19 pandemic. Inclusion criteria were the following: (a) previous diagnosis of NT1 according to international criteria (American Academy of Sleep Medicine, 2014); (b) agreeing to a telephone consultation instead of postponing the follow‐up assessment until after the emergency end; (c) having been under stable pharmacological treatment for at least one year; and (d) being already enrolled in an ongoing prospective study approved by the local ethics committee (Comitato Etico Interaziendale Bologna‐Imola, CE‐BI, Prot. Num. 17009). According to clinical practice, all patients were asked about body weight, current pharmacological treatment, current habitual sleep schedules (night‐time sleep time, frequency of daytime napping), presence and severity of core narcolepsy symptoms (including subjective sleepiness according to the Italian version of the Epworth Sleepiness Scale, ESS) (Vignatelli et al., 2003), occurrence and frequency of automatic behaviors assessed on a Likert scale (<1/year; between 1/year and 1/month; between 1/month and 1/week; between 1/week and 1/day; >1/day), cataplexy, sleep paralyses, hypnagogic hallucinations, presence of nightmares, disturbed nocturnal sleep, and behavioral (nocturnal sleep hygiene, daytime planned naps), pharmacological treatment, and current working status (patients with maintained working schedule—"unchanged"; forced to " working/studying at home"; and losing their job—"lost occupation"). The same prelockdown data were collected from patients' records considering the previous follow‐up assessment (6 months before in our clinical routine), along with age at NT1 symptoms onset and age at diagnosis.

Patients' characteristics were analyzed by descriptive statistics (mean and standard deviation or percentage for numerical or categorical variables, respectively). Patients' clinical characteristics were compared between groups with Kruskal–Wallis ANOVA or chi‐square tests for numerical and categorical variables, respectively. Prelockdown and during‐lockdown data were compared by means of nonparametric within‐subjects comparisons (McNemar's test) separately for each subgroup defined on the basis of current working schedules (i.e., unchanged, working/studying at home, and lost occupation).

## 3 RESULTS

### 3.1 Population

During the study period, there were 94 scheduled follow‐up visits at the Outpatient Clinic for Narcolepsy. According to inclusion criteria, the study was proposed to 53 patients. Three patients did not accept to participate in the study (two preferred to postpone the clinical assessment after the COVID‐19 pandemic emergency, and one was unable to perform the visit because of work duties). We therefore collected data about 50 patients (40% males) with a mean age of 35.0 ± 19.4 years at assessment, of 28.6 ± 19.2 at diagnosis, and of 20.8 ± 15.8 at NT1 symptoms onset. Patients reported the following occupational status: employed (30%); self‐employed (6%); student (36%); housewife (6%); retired (8%); and unemployed (14%). Patients were prescribed behavioral (90%) and pharmacological (88%) treatments, the latter including sodium oxybate (76%), modafinil (40%), pitolisant (20%), venlafaxine (14%), and imipramine (6%).

### 3.2 Clinical data and comparisons among different working schedule groups

Twenty patients (40%) did not undergo any change in the working schedule because of the lockdown, while 22 (44%) and 8 (16%) reported working/studying at home or having lost their job, respectively. Patients working/studying at home had lower BMI (23 vs. 28/29) and were younger both at time of evaluation also and at time of diagnosis. Indeed, they were more frequently students, whereas a higher proportion of unemployment was present in the patients with “unchanged scheduled.” Conversely, the three groups did not differ for gender distribution, nor for ESS score, or any NT1 symptoms severity at baseline assessment (Table 1).

**TABLE 1:** Clinical features of NT1 patients subgroups with between‐groups (baseline) and within‐group comparisons

	Unchanged schedule, *n* = 20				Within‐subjects comparison	Working/studying at home, *n* = 22				Within‐subjects comparison	Lost occupation, *n* = 8				Within‐subjects comparison	Baseline between‐groups comparison
	PRE		DURING		*p*‐VALUE	PRE		DURING		*p*‐VALUE	PRE		DURING		*p*‐VALUE	*p*‐VALUE
	*M*/%	*SD*	*M*/%	*SD*		*M*/%	*SD*	*M*/%	*SD*		*M*/%	*SD*	*M*/%	*SD*		
Male (%)	35.00					36.40					62.50					.365
Age at evaluation (year)	49.45	20.05				20.86	7.54				37.75	11.31				**.000**
NT1 onset age (year)	29.26	19.19				10.77	3.70				28.38	12.45				**.000**
NT1 diagnosis age (year)	42.84	19.68				14.27	7.00				34.25	12.02				**.000**
BMI (Kg/m^2^)	28.91	6.47	29.61	7.75	.239	23.39	5.94	23.80	5.81	**.006**	28.68	5.22	29.47	6.42	.173	**.003**
ESS score (*n*)	12.05	5.54	10.95	4.86	.076	10.86	4.27	8.68	4.19	**.026**	10.86	4.85	8.38	3.42	.098	.916
Nocturnal sleep duration (h)	7.58	2.48	7.32	2.29	.273	7.95	1.03	8.34	1.42	**.028**	7.75	1.07	8.10	1.27	.109	.920
Schooling																
Primary school (%)	5.6					9.1					0.0					**.004**
Middle school (%)	22.2					77.3					71.4					
High school (%)	50.0					0.0					28.6					
University degree (%)	22.2					13.6					0.0					
Employment status																
Employed	30.0					18.2					62.5					**.000**
Self‐employed	0					0.0					37.5					
Student	0.0					81.8					0.0					
Housewife	15.0					0.0					0.0					
Retired	20.0					0.0					0.0					
Unemployed	35.0					0.0					0.0					
Behav. treatment frequency																
Never	15.0		20.0		.593	0.0		4.5		**.050**	25.0		12.5		.059	.145
On demand	5.0		10.0			27.3		4.5			25.0		0.0			
1/day	45.0		40.0			45.5		36.4			50.0		50.0			
>1/day	25.0		30.0			27.3		54.5			0.0		37.5			
Automatic behavior frequency																
<1/year	75		70.0		.317	81.8		77.3		.655	100.0		100.0		1.000	.724
1/year–1/month	5.0		5.0			0.0		0.0			0.0		0.0			
1/month–1/week	5.0		10.0			0.0		13.6			0.0		0.0			
1/week–1/day	5.0		15.0			4.5		4.5			0.0		0.0			
>1/day	0.0		0.0			0.0		4.5			0.0		0.0			
Cataplexy frequency																
<1/year	35.0		40.0		.792	36.4		36.4		.236	25.0		37.5		.336	.664
1/year–1/month	15.0		15.0			13.6		9.1			12.5		12.5			
1/month–1/week	25.0		10.0			31.8		22.7			12.5		25.0			
1/week–1/day	20.0		25.0			4.5		22.7			37.5		25.0			
>1/day	5.0		10.0			9.1		9.1			12.5		0.0			
Sleep paralysis frequency																
<1/year	65.0		75.0		.317	86.4		90.9		.317	75		75.0		.655	.518
1/year–1/month	5.0		10.0			4.5		9.1			0.0		0.0			
1/month–1/week	15.0		5.0			4.5		0.0			0.0		12.5			
1/week–1/day	5.0		5.0			0.0		0.0			12.5		12.5			
>1/day	5.0		5.0			0.0		0.0			0.0		0.0			
Hypnagogic hallucination frequency																
<1/year	80.0		75.0		.564	77.3		72.7		.317	62.5		75.0		.317	.447
1/year–1/month	0.0		10.0			4.5		4.5			0.0		0.0			
1/month–1/week	15.0		5.0			9.1		9.1			0.0		0.0			
1/week–1/day	0.0		5.0			4.5		4.5			0.0		12.5			
>1/day	5.0		5.0			4.5		4.5			25.0		12.5			
Disturbed nocturnal sleep (%)	15.0		45.0		**.014**	4.5		18.2		.180	12.5		25.0		.564	.512
Nightmares (%)	15.0		25.0		.317	13.6		18.2		.317	12.5		12.5		1.000	.983

Note

In bold, statistically significant result: *p* < .05.

Abbreviations: Behav, behavior; BMI, body mass index; ESS, Epworth Sleepiness Scale; M/%, mean/percentual; NT1, narcolepsy type 1; *SD*, standard deviation.

The within‐group comparison according to work schedule changes showed that patients with unchanged occupational condition had a significant increase in disturbed nocturnal sleep complaints (from 15% to 40%), whereas patients who lost occupation did not show any significant clinical change. Conversely, NT1 patients working/studying at home showed a significant increase in the reported nocturnal sleep time (from 7.9 to 8.3 hr), in the frequency of daytime napping (54.4% vs. 27.3 reporting multiple daytime scheduled naps at current and previous evaluations, respectively), and a significant decrease in daytime sleepiness intensity (ESS score from 10.8 to 8.7). Patients working/studying at home showed a slight, but statistically significant, increase in their body mass index from 23.4 to 23.8; Table 1).

## 4 DISCUSSION

We reported behavioral and clinical changes related to the COVID‐19 pandemic lockdown in a consecutive cohort of NT1 patients under stable, long‐term, pharmacological treatment. We found that patients who continued their usual working schedule suffered more frequently from nocturnal awakenings, and both patients with unchanged working schedule and those who lost their occupation because of the COVID‐19 pandemic did not show significant NT1 symptoms severity changes. Conversely, NT1 patients working/studying at home seem to have enhanced behavioral strategies (increasing the duration of nocturnal sleep time and the frequency of daytime napping) and showed a significant decrease in daytime sleepiness (at ESS) paralleled by an increase in BMI. Our finding of increased nocturnal sleep time in NT1 patients working/studying at home, also taking into account the high representation of students in our clinical population, is in line with studies exploring the impact of lockdown on sleep patterns among young adults, showing later sleep timings and increased nocturnal sleep time (Altena et al., 2020; Cellini et al., 2020; Kaparounaki et al., 2020). Our data also suggested that, in patients with NT1 under stable long‐term pharmacological treatment, the possibility to self‐manage daytime activities allowed more daytime planned naps. To date, only few experimental data proved the efficacy of napping to reduce daytime sleepiness in NT1, and the authors reported that patients who applied a regular nocturnal sleep hygiene combined, if applicable, with daytime napping had a significant amelioration of narcolepsy symptoms compared with those making only daytime napping (Rogers et al., 2001). The above findings and our clinical observation are in line with the common medical attitude to integrate pharmacological prescriptions with the recommendation to apply behavioral strategies to reduce daytime sleepiness intensity and the need of drugs (Franceschini et al., 2020). However, this approach is not currently supported by strong scientific evidence (Krahn et al., 2015) and relies mostly on expert opinions. We also found that patients working/studying at home increased their BMI, differently from the other two patient groups. However, patients working/studying at home presented with a significantly lower BMI before the lockdown compared with the other two groups. This finding may reflect the impact of reduced physical activity during the lockdown (not assessed in our clinical routine) and may well correlate with the evidence of higher BMI among patients with NT1 who do not regularly practice sport also at a young age (Filardi et al., 2018). In parallel, the association between obesity and NT1 is well established (Bassetti et al., 2019), and therefore, the evidence of a high BMI in both NT1 patients not changing their working schedule and those losing occupation is in line with the literature, as well as the rapid, albeit slight, increase in body weight during confinement.

The finding of increased nocturnal awakenings in patients maintaining their current working schedule is consistent with the poorer sleep quality reported by the general population during the lockdown (Brooks et al., 2020; Cellini et al., 2020; Kaparounaki et al., 2020). Indeed, NT1 is also intrinsically characterized by disturbed nocturnal sleep at all ages (Maski et al., 2020; Roth et al., 2013), and an increased level of stress and anxiety (not quantified in our clinical observation) may underlie the worsening of nocturnal sleep continuity despite unchanged, long‐term treatment. The higher proportion of unemployment among them could reflect the longer disease duration and diagnostic delay, highlighting that a early diagnosis and treatment could prevent the detrimental disease's burden in psychosocial aspect of life (Ingravallo et al., 2012; Maski et al., 2017). The awareness of the disease at a young age could provide the opportunity to make educational and job choices more suited to patients' needs.

This report has some limitations. First, we assessed treatment and symptom severity during the lockdown as during routine visits in order to allow a direct comparison with previously available clinical data, but without considering other psychological aspects of potential importance in this peculiar period. Indeed, we did not report information about different housing situation that could influence the perception of lockdown restrictive measures, thus calling for further larger scale studies on the relation between sleep–wake habits, working schedule, and symptoms control in narcolepsy. Second, we focused on patients under stable long‐term treatment in order to allow us to make comparisons with prelockdown data. Given the possibility of changes in pharmacological compliance, a more complex picture including interactions between working schedule, compliance with treatment, sleep patterns, and symptom severity may be addressed by larger scale studies. Third, our patient groups were not age‐matched, and the small sample size does not allow a multivariate statistical analysis to take into account the effect of age.

To conclude, we found that young NT1 patients working/studying at home modified their sleep–wake patterns, improving sleep hygiene and behavioral interventions. This may have reduced daytime sleepiness, but also determined a slight, but significant, increase in body weight. This finding, in light of the few data supporting the utility of behavioral therapy (Franceschini et al., 2020), should promote further research on the efficacy of behavioral interventions in NT1, focusing on sleep hygiene, scheduled napping, and physical activity.

## CONFLICTS OF INTEREST

The authors declare no potential conflicts of interest.

## AUTHOR CONTRIBUTION

E.Postiglione and F.Pizza with the contribution of the other co‐authors designed the study, collected, analyzed, and interpreted the data, and drafted and revised the manuscript. F.Ingravallo, L.Vignatelli, U.Pagotto, and G.Plazzi conceptualized the work and revised the manuscript. M.Filardi, A.Mangiaruga, E.Antelmi, M.Moresco, and C.Oriolo collected the data.

### Peer Review

The peer review history for this article is available at https://publons.com/publon/10.1002/brb3.1955.

## Data Availability

The data that support the findings of this study are available from the corresponding author upon reasonable request.

## References

[brb31955-bib-0001] Altena, E. , Baglioni, C. , Espie, C. A. , Ellis, J. , Gavriloff, D. , Holzinger, B. , Schlarb, A. , Frase, L. , Jernelöv, S. , & Riemann, D. (2020). Dealing with sleep problems during home confinement due to the COVID‐19 outbreak: Practical recommendations from a task force of the European CBT‐I Academy. Journal of Sleep Research, e13052. 10.1111/jsr.13052. Epub Ahead Of Print. 32246787

[brb31955-bib-0002] American Academy of Sleep Medicine (2014). International classification of sleep disorders ‐ third edition (ICSD‐3). American Academy of Sleep Medicine.

[brb31955-bib-0003] Bassetti, C. L. A. , Adamantidis, A. , Burdakov, D. , Han, F. , Gay, S. , Kallweit, U. , Khatami, R. , Koning, F. , Kornum, B. R. , Lammers, G. J. , Liblau, R. S. , Luppi, P. H. , Mayer, G. , Pollmächer, T. , Sakurai, T. , Sallusto, F. , Scammell, T. E. , Tafti, M. , & Dauvilliers, Y. (2019). Narcolepsy ‐ clinical spectrum, aetiopathophysiology, diagnosis and treatment. Nature Reviews Neurology, 15(9), 519–539. 10.1038/s41582-019-0226-9 31324898

[brb31955-bib-0004] Brooks, S. K. , Webster, R. K. , Smith, L. E. , Woodland, L. , Wessely, S. , Greenberg, N. , & Rubin, G. J. (2020). The psychological impact of quarantine and how to reduce it: Rapid review of the evidence. Lancet, 395(10227), 912–920. 10.1016/S0140-6736(20)30460-8 32112714 PMC7158942

[brb31955-bib-0005] Cellini, N. , Canale, N. , Mioni, G. , & Costa, S. (2020). Changes in sleep pattern, sense of time and digital media use during COVID‐19 lockdown in Italy [published online ahead of print, 2020 May 15]. Journal of Sleep Research, e13074. 10.1111/jsr.13074. Epub Ahead Of Print. 32410272 PMC7235482

[brb31955-bib-0006] Filardi, M. , Pizza, F. , Antelmi, E. , Pillastrini, P. , Natale, V. , & Plazzi, G. (2018). Physical activity and sleep/wake behavior, anthropometric, and metabolic profile in pediatric narcolepsy type 1. Frontiers in Neurology, 9, 707. 10.3389/fneur.2018.00707 30197622 PMC6117389

[brb31955-bib-0007] Franceschini, C. , Pizza, F. , Antelmi, E. , Folli, M. C. , & Plazzi, G. (2020). Narcolepsy treatment: Pharmacological and behavioral strategies in adults and children. Sleep and Breathing, 24(2), 615–627. 10.1007/s11325-019-01894-4 31290083

[brb31955-bib-0008] Ingravallo, F. , Gnucci, V. , Pizza, F. , Vignatelli, L. , Govi, A. , Dormi, A. , Pelotti, S. , Cicognani, A. , Dauvilliers, Y. , & Plazzi, G. (2012). The burden of narcolepsy with cataplexy: How disease history and clinical features influence socio‐economic outcomes. Sleep Medicine, 13(10), 1293–1300. 10.1016/j.sleep.2012.08.002 23026503

[brb31955-bib-0009] Kaparounaki, C. K. , Patsali, M. E. , Mousa, D. V. , Papadopoulou, E. V. K. , Papadopoulou, K. K. K. , & Fountoulakis, K. N. (2020). University students' mental health amidst the COVID‐19 quarantine in Greece [published online ahead of print, 2020 May 19]. Psychiatry Research, 290: 113111. 32450416 10.1016/j.psychres.2020.113111 PMC7236729

[brb31955-bib-0010] Krahn, L. E. , Hershner, S. , Loeding, L. D. , Maski, K. P. , Rifkin, D. I. , Selim, B. , & Watson, N. F. (2015). Quality measures for the care of patients with narcolepsy. Journal of Clinical Sleep Medicine, 11(3), 335. 10.5664/jcsm.4554 25700880 PMC4346654

[brb31955-bib-0011] Li, C. , Romagnani, P. , von Brunn, A. , & Anders, H.‐J. (2020). SARS‐CoV‐2 and Europe: Timing of containment measures for outbreak control. Infection, 48(3). 10.1007/s15010-020-01420. (published online April 9.) PMC7142270 32274649

[brb31955-bib-0012] Maski, K. , Pizza, F. , Liu, S. , Steinhart, E. , Little, E. , Colclasure, A. , Diniz Behn, C. , Vandi, S. , Antelmi, E. , Weller, E. , Scammell, T. E. , & Plazzi, G. (2020). Defining disrupted nighttime sleep and assessing its diagnostic utility for pediatric narcolepsy type 1 [published online ahead of print, 2020 Apr 7]. Sleep, 43(10), zsaa066. 10.1093/sleep/zsaa066 32253429 PMC7551299

[brb31955-bib-0013] Maski, K. , Steinhart, E. , Williams, D. et al (2017). Listening to the patient voice in narcolepsy: diagnostic delay, disease burden, and treatment efficacy. Journal of Clinical Sleep Medicine, 13(3), 419–425. 10.5664/jcsm.6494. Published 2017 Mar 15 27923434 PMC5337589

[brb31955-bib-0014] Raggi, A. , Plazzi, G. , & Ferri, R. (2019). Health‐related quality of life in patients with narcolepsy: A review of the literature. The Journal of Nervous and Mental Disease, 207(2), 84–99. 10.1097/NMD.000000000000091 30672873

[brb31955-bib-0015] Rogers, A. E. , Aldrich, M. S. , & Lin, X. (2001). A comparison of three different sleep schedules for reducing daytime sleepiness in narcolepsy. Sleep, 24(4), 385–391. 10.1093/sleep/24.4.385 11403522

[brb31955-bib-0016] Roth, T. , Dauvilliers, Y. , Mignot, E. , Montplaisir, J. , Paul, J. , Swick, T. , & Zee, P. (2013). Disrupted nighttime sleep in narcolepsy. Journal of Clinical Sleep Medicine, 9, 955–965. 10.5664/jcsm.3004 23997709 PMC3746724

[brb31955-bib-0017] Vignatelli, L. , Plazzi, G. , Barbato, A. , et al. (2003). Italian version of the Epworth sleepiness scale: External validity. Neurological Sciences, 23(6), 295–300. 10.1007/s100720300004. 12624716

